# Retiring Address of Dr. S. B. Palmer, Pres. of the Central New York Dental Society

**Published:** 1865-07

**Authors:** John F. Johnston

**Affiliations:** Corresponding and Recording Secretary


### RETIRING ADDRESS OF DR. S. B. PALMER,

PRESIDENT OF THE CENTRAL NEW YORK DENTAL SOCIETY.

*Friends and Professional Brethren*,—Custom might sanction, if not call for remarks from a retiring officer; yet I would take no advantage of such an opportunity to occupy one moment of your time.

We have convened to consider the best interests of our Association, to stimulate its members to higher aspirations and more vigorous action, and to discuss subjects beneficial to those requiring treatment at our hands.

There is not a member in our association, who can truthfully say, I have nothing to do. There is a duty to be performed which no dentist can excuse himself from, the duty of improvement; he owes it to self, to his profession, and in the highest degree to his patient.

We would contribute our mite towards rearing an observatory, from which to view the work accomplished by the energetic and persevering members of the profession. We flatter ourselves that to-day we occupy a position higher than ever before. With the telescope of the dental journals, we survey not only the American continent, but all Europe, and behold the progress everywhere attained by the liberal exchange of science and philosophy.

It is not our purpose to give a review of dental literature, nor even a summary of late inventions or improvements, as the ever welcome journals afford ample means for obtaining such information; yet we feel proud to represent a profession so awake to the advancement of science, so desirous of ameliorating the condition of suffering humanity.

We will discuss the subject of improvement at large, and look to our own Association. Will our annual report to the American Dental Association show that we are contributing our portion to progress? Have we sought every means for improvement through dental publications? Have we adopted new theories, or been benefitted by modern discoveries? Have we striven to make each operation better than the preceding one? Have our decisions been rendered for the greatest good of the patient, rather than for any pecuniary motive of our own? then have we been improving ourselves, and enlightening others. On the contrary, if we have been satisfied with former attainments and are still guided by the stereotyped directions of former days, fearing to advance in science, or fees for the same, then professional advancement has no charms for us, and we have no claims for the honor of progress.

There are three great opportunities for improvement, Dental Colleges, Dental Literature, and Dental Associations; three in name, one in purpose, auxiliaries to each other. Dental Colleges are indispensable, aside from the opportunities there afforded to the dental student to complete his professional studies, the profession receive from them the true standard of dental education. While a few dental students, from private study and private teaching might receive as thorough instruction, others less fortunate would receive but imperfect teaching upon a limited number of subjects. We rejoice that we have such a means of dental instruction.

Dental literature is another and more general means of diffusing knowledge, bringing the latest discoveries and improvements within the reach of all; home instruction without a master; the class book from which to learn lessons; while dental associations are the meetings for recitation, an examination of former theories and observations by the test of practice and demonstration.

Who will say on the present occasion like the truant schoolboy, "not prepared to-day." As individuals we have a work to perform for improvement.

We lately saw a war map which had been published but a short time, entitled, "The Rebellion as it was, The Rebellion as it is;" a dark shade covered a portion of the contested territory; but a few months have elapsed since that map was engraved, and now we want another to show the advancement of the Union cause, showing no dark shade, no Rebellion.

So with the progress of the dental cause, though less rapid; so great are the changes that the report of last year's transactions only shew dentistry "as it was."

To the American Dental Association we owe much for the establishment of local societies, the addition to our libraries of those valuable volumes of its transactions; and not less, the improved social condition of dentists brought under its influence.

A number of those present to-day are members of that body, and now is offered another opportunity to become members, delegates are to be commissioned to represent our Society in the next meeting of the Association. One of the advantages gained by associated gatherings is the breaking down of prejudices which creep into some minds, to make them believe they know all that is worth knowing, or others to think they know but little, and entertain fears that truth revealed would show "Dentistry as it was."

We believe that our present attainments have given to our profession character, which will rank favorably with Medicine, Law or Divinity. Professional, character is formed by individual character, their standing for virtue and morality become the standard for the body they represent.

These qualities may be associated, and improved as well as those of skill or invention.

While we do not attempt to mark out the precise conduct to be pursued towards our patients, still there is a right way, and the nearer we get in the line of duty, the closer will our manipulations and the results, resemble each other.

There is manifestly a division of talent in our profession, the higher and lower. Nature may have done much in assigning us to one or the other, in giving or withholding, her gift of skill adapted to our necessity; yet industry, and determined perseverance does quite as much towards success.

In order to become seccessful there must be a higher than a dollar and cent view of the operation. We know that we are presenting a familiar subject, yet there is an excuse for so doing. Progress is our motto. Keep it before the Profession our watchword, so long as there is such a reckless sacrifice of valuable organs. Teeth are daily doomed to the forceps, which by our art could be preserved for years of usefulness.

This distruction is not to be charged to the ignorant only, but to all who conform to the desires, and yield to the requests of misguided or ignorant patients.

To be successful we must sustain a good reputation, and maintain the character of the Profession, which can not be done by cheap operations. Price is king, labor and material are legal subjects.

In all the cases presented for the Dentist decision there is a best way, still no minute rule can be layed down to govern in such decisions. Time and opportuuity to perform an operation, willingness to submit to proper treatment, and ability to pay for the same, are influences likely to swerve the well designing, from the right way. Here comes the division of talent, firmly adheiing to right and justice, consulting the best interest of the patient as well as the operator, places one on the ascending grade, while from any circumstance, the operator is induced to receive a mere pitance and conform to the unjust elements of ignorant or penurious patients establishes him in the lower grade of talent.

When a Dentist is besought to use the forceps, against his convictions, let him call to mind that injunction in Holy Writ "Remember thy Creator" and by acts, acknowledge him to be a superior Dentist. Let Nature's work be *preserved* rather than destroyed.

With due respect to the patient the dentist should insist that his knowledge is superior to their's in matters relating to the teeth. The patient has a right to demand a first class operation, if other is given with means at hand far greater knowlenge, and higher skill, that is an unjust act. It becomes the duty of the operator to enlighten his patient in all particulars relating to care, cleanliness and the preservation of their teeth, not to calculate which will afford him the highest revenue, the use of the plugger or forceps.

Now gentleman that the excitement of war has passed away and we behold some of our Profession returning from the military ranks—to join us, may we with them act vigorously in driving back the dark shadows of empiricism and ignorance, until by the close of another year we shall need a new record of events to show dentistry as it was and dentistry as it may be.

### Proceedings of Societies.

---

The Indiana State Dental Association, met according to announcement in the rooms of the Indianapolis Medical Association, on the last Tuesday in June, 1865. Quite a large number of the profession were present, and a very interesting and instructive session was the result.

The Officers for the ensuing term are:

*President*—Dr. Joseph Richardson, Terre Haute.

1*st.* *Vice-President*—Dr. P. G. C. Hunt, Indianapolis.

2*nd.* " " Dr. C. C. Burgess, Indianapolis.

3*d.* " " Dr. P. W. Morris, Greensburg.

*Corresponding and Recording Secretary*—Dr. John F. Johnston, Indianapolis.

*Treasurer*—Dr. G. A. Wells, Indianapolis.

Delegates to American Dental Association.

Dr. D. M. Weld, Terre Haute.

" C. C. Burgess, Indianapolis.

" M. Wells, Indianapolis.

" Joseph Richardson, Terre Haute.

" P. G. C. Hunt, Indianapolis.

Essayests for the next meeting, Drs. D. M. Weld, C. C. Burgess and Merit Wells.

In consequence of no reporter being present, it is quite impossible for the Secretary to give to the profession, that which would most interest them, viz; a report of the discussions. It is hoped that at the next meeting this omission will be provided for.

Preferring not to annoy either the Journals or the Profession with dry business details; he will only add that he thinks the Profession in Indiana will find it to their interest to give more particular attention to the interest of their State Association, than they have done heretofore. He is happy however, to announce that the Association is now upon a permanent footing, and from the great interest manifested by those present at this meeting, he predicts a very full attendance at the next, which will take place at the City of Indianapolis, on the the last Tuesday in June, 1866, and to which the Profession throughout the State, in particular, are cordially invited, and any others that can come.

JOHN F. JOHNSTON,
*Corresponding and Recording Secretary.*

